# Interventions for the Prevention and Management of Nipple Trauma in Breastfeeding Women: A Systematic Review

**DOI:** 10.3390/healthcare14111546

**Published:** 2026-06-02

**Authors:** Simela Kirimlidou, Maria Dagla, Ermioni Palaska, Kleanthi Gourounti, Angeliki Sarella, Eirini Orovou, Maria Iliadou

**Affiliations:** 1 Midwifery Department, School of Health and Care Sciences, University of West Attica, 12243 Athens, Greece; melikirimlidou@gmail.com (S.K.); mariadagla@uniwa.gr (M.D.); epalaska@uniwa.gr (E.P.); kgourounti@uniwa.gr (K.G.); asare@uniwa.gr (A.S.); 2 Department of Midwifery, University of Western Macedonia, 50200 Ptolemaida, Greece; eorovou@uowm.gr

**Keywords:** breastfeeding, nipple trauma, nipple pain, breastfeeding interventions, nipple trauma prevention

## Abstract

**Highlights:**

**What are the main findings?**

- Topical application of natural products and nipple shields can help reduce pain and support healing, although effectiveness varies.
- Education and professional support improve breastfeeding technique, experience, and continuation.
- A combined, individualized approach appears most beneficial, as no single intervention is effective for all cases.

**What are the implications of the main findings?**

- Interventions should be personalized based on the mother’s needs, nipple condition, and breastfeeding challenges.
- Integrating topical, technical, and educational support is key to improving pain relief, nipple healing, and breastfeeding outcomes.

**Abstract:**

**Background/Objectives:** Nipple trauma is one of the most common complications during breastfeeding and may lead to pain, discomfort, and the premature cessation of lactation. This systematic review aimed to synthesize and critically appraise recent evidence (2020–2026), reflecting current clinical practices and emerging interventions, for the prevention and management of nipple trauma in breastfeeding women. The primary emphasis was on pain reduction and healing with a secondary consideration of their impact on breastfeeding continuation and duration. **Methods:** The review was conducted according to the PRISMA guidelines and included a literature search in the PubMed and Scopus databases. Primary research studies published between 2020 and 2026 focusing on interventions in breastfeeding women experiencing nipple trauma or nipple pain were included. A total of 22 studies met the inclusion criteria. **Results:** The interventions included the topical applications of natural products such as olive oil, expressed breast milk, coconut oil, beeswax-based products, and lanolin, nipple shields, and educational interventions focusing on correct breastfeeding techniques. The findings suggest that several of these interventions may reduce nipple pain and promote the healing of nipple trauma, while breastfeeding education and professional support appear to improve breastfeeding experience and continuation. **Conclusions:** The heterogeneity of the included studies highlights the need for further well-designed clinical research.

## 1. Introduction

Breastfeeding is widely recognized as a fundamental biological process of the perinatal period and as a basic public health practice. International health organizations recommend initiating breastfeeding within the first hour after birth and maintaining exclusive breastfeeding for the first six months of life with continued breastfeeding alongside the introduction of appropriate complementary foods thereafter [1]. Understanding breastfeeding requires knowledge of the mechanisms of milk production and secretion as well as the anatomy and histology of the breast, particularly the nipple–areola complex. This area is the critical point of contact between mother and infant, allowing both milk transfer and the neuroendocrine activation of lactation [2]. The nipple and areola constitute a specialized functional unit that plays a central role in breastfeeding. Histologically, the nipple is covered by a keratinized stratified squamous epithelium and contains smooth muscle fibers arranged in circular and longitudinal patterns, connective tissue, and the terminal openings of the milk ducts. The epidermis in this region, under normal conditions, acts as both a mechanical and biochemical barrier, protecting against water loss, microbial invasion, and chemical irritation [3]. These smooth muscle fibers allow the nipple to change shape and protrude in response to mechanical or thermal stimuli. Importantly, the nipple–areola complex is highly innervated, allowing sensory stimulation during the infant’s suckling to activate neuroendocrine responses necessary for lactation [2].

The integrity of nipple skin depends on multiple factors including hydration, pH homeostasis, the level of transepidermal water loss (TEWL), and the quantity and quality of surface lipids [4]. Maintaining these parameters is particularly important during lactation. During this period, the nipple and areola are exposed to repeated mechanical stress and continuous contact with fluids such as breast milk, sweat, and infant saliva. These factors may alter the epidermal microenvironment and affect the functional integrity of the skin. It has been noted that epithelial overhydration and prolonged moisture exposure may predispose the tissue to moisture-associated skin damage, increasing the vulnerability of the epidermis to microfissures or disruptions that may later develop into more visible skin lesions [5]. In this context, the nipple should not be regarded merely as the outlet of the milk ducts but rather as a functionally active and sensitive skin tissue in which the balance between moisture and lipid protection represents a critical biological factor.

Particular importance within this cutaneous microenvironment is attributed to the glands of the areola, which are known as Montgomery glands or tubercles. These are sebaceous–apocrine glands located on the surface of the areola that exhibit characteristic hypertrophy during pregnancy and lactation, which is a change that is considered an adaptive mechanism supporting the function of the nipple–areola complex during breastfeeding [6]. Their secretions are described as lipid substances that may contribute to the natural lubrication of the nipple and potentially create a protective film on its surface, thereby supporting the skin’s resistance to friction and mechanical irritation. In addition, it has been suggested that the secretions of Montgomery glands may also play a chemosensory and behavioral role, as they contain volatile compounds capable of eliciting responses in the newborn and supporting the infant’s innate ability to orient toward the nipple [7]. Thus, Montgomery glands are theoretically associated both with the biological protection of the nipple and with aspects of neonatal behavior related to the initiation and effectiveness of breastfeeding.

Due to its structure and function, the nipple is particularly susceptible to mechanical loads during breastfeeding, as it undergoes repeated stretching, compression, and friction. The integrity of the nipple epidermis, tissue elasticity, skin hydration level, and local blood supply are factors associated with the resilience of this area to microtrauma. Furthermore, the functional coordination between the nipple and the infant’s mouth—through the infant’s latch and the coordinated movements of the tongue and jaws—is essential for effective and comfortable milk transfer and for maintaining effective breastfeeding without discomfort. The presence of nipple pain, especially when persistent or worsening, may alter the maternal neurohormonal response and influence the breastfeeding experience both physically and psychologically, highlighting the importance of understanding the physiological and anatomical parameters governing this process [8].

Nipple pain and trauma are multifactorial conditions that extend beyond maternal anatomical and physiological factors. Infant-related variables play a critical role, including incorrect positioning and attachment [9], oral anatomical variations (such as ankyloglossia) [10], and the intraoral vacuum dynamics [11] during breastfeeding. Suboptimal latch or ineffective sucking mechanics may increase mechanical stress on the nipple, leading to tissue damage and impaired healing.

Additionally, the nipple and areola region present particular characteristics in terms of skin morphology and microbial flora. As a skin area with glands, folds, and increased moisture, it may represent a site where dermatological conditions or irritations may occur. Consequently, the diagnostic approach to the nipple requires differentiation between normal physiological changes, benign findings, and pathological conditions. Understanding normal structures and variations—such as nipple inversion, congenital anomalies, or benign lesions—is essential in order to avoid misinterpretations or interventions that could potentially affect the course of lactation [3].

Based on the above, it becomes clear that breastfeeding is a complex phenomenon grounded in precise anatomical structures and multifaceted physiological mechanisms. Fundamental knowledge of the breast, the nipple, and the neuroendocrine regulation of lactation constitutes essential theoretical background for the scientific understanding of issues related to the breastfeeding experience, the maintenance of lactation, and the factors that may influence it.

Also, prior systematic reviews [12,13,14,15,16] have examined interventions aimed at the prevention and management of nipple trauma during breastfeeding, generally reporting a wide range of approaches with variable effectiveness and considerable heterogeneity in study design and outcome measures. Many of these reviews include primary studies conducted prior to 2020, combining interventions and clinical practices that might not fully represent contemporary breastfeeding support strategies. In addition, the recent development of new products and evolving clinical guidance in recent years highlight the need for an updated synthesis of evidence.

Therefore, the present systematic review focuses especially on studies published between 2020 and 2026, aiming to provide a targeted and up-to-date evaluation of recent clinical interventions for nipple trauma. This approach is intended to complement existing reviews by emphasizing contemporary evidence and its relevance to current clinical practice. Primary emphasis is given on pain reduction and healing with a secondary consideration given to how these factors may affect breastfeeding continuation and duration.

## 2. Materials and Methods

### 2.1. PICO Eligibility Criteria

In breastfeeding women, regardless of age, mode of delivery, or care setting (P), how do interventions aimed at preventing and/or managing nipple trauma (I) compare to usual care, no intervention, placebo, or alternative interventions (C) in terms of reducing pain intensity, improving or healing nipple trauma, and supporting breastfeeding continuation or duration (O)?

For clarity, “standard interventions” in the context of nipple trauma typically include established first-line measures such as the application of topical based treatments, breastfeeding education and technical issues. Also, this review uses the term “alternative interventions” to describe non-routine or adjunctive approaches that extend beyond standard care, including device-based and other interventional strategies.

Table 1 summarizes the key elements of the PICO framework.

### 2.2. Literature Search, Study Selection and Data Synthesis

A systematic search was conducted in PubMed and Scopus to identify all relevant studies published between 2020 and 2026 to capture the most updated evidence reflecting current breastfeeding practices and interventions to nipple trauma management. The strategy combined keywords and subject headings related to breastfeeding and nipple trauma, including “breastfeeding”, “lactation”, “nipple trauma”, “sore nipples”, “cracked nipples”, “nipple pain”, “intervention”, “prevention”, and “treatment”. Boolean operators (AND, OR) were used to maximize sensitivity. Full reference lists of included studies were also manually screened to identify additional relevant publications.

**Figure 1:**
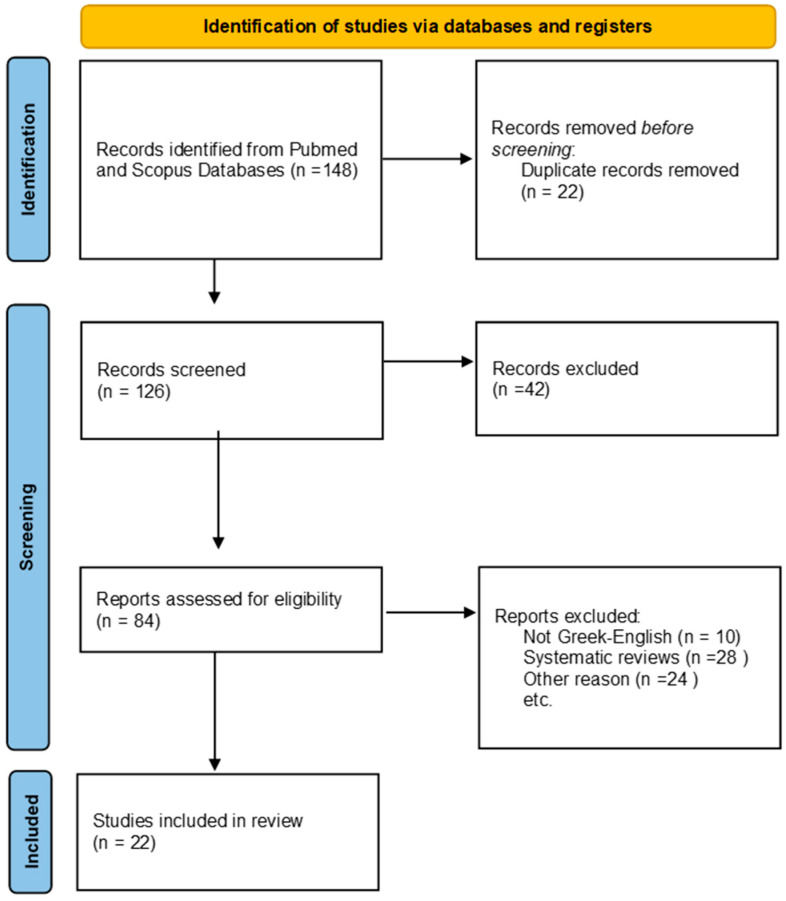
Flow diagram of articles included in the systematic review.

Study selection followed a PRISMA-based, multi-stage process. The search was limited to studies published within the past six years and identified 148 potential records from the above-mentioned databases. After removing 22 duplicates, titles and abstracts were screened, resulting in the exclusion of 42 studies. A total of 84 full-text articles were assessed for eligibility. Following full-text evaluation, 62 studies were excluded based on predefined criteria, leaving 22 studies included in the final review. The study selection process is presented in the PRISMA flow diagram (Figure 1).

A meta-analysis was not conducted due to the substantial heterogeneity in interventions and outcome variability, which limited the comparability of results and precluded quantitative synthesis. Therefore, a narrative synthesis approach was adopted.

Studies were excluded if they did not involve breastfeeding women or were unrelated to nipple trauma or pain; did not assess a specific intervention; lacked primary research data (e.g., reviews, letters, commentaries, case reports); were not available in full text; were published in languages other than English or Greek; or involved animal subjects.

Data were extracted using a standardized form by two independent researchers (S.M.K, M.I) and cross-verified for accuracy. Extracted information included study authors, type, year, sample size, and outcomes related to nipple trauma interventions. A narrative synthesis summarized the study design, assumptions, intervention effects, and overall methodological quality.

### 2.3. Methodological Quality Assessment

The methodological quality and potential risk of bias of the included studies were evaluated using the Mixed Methods Appraisal Tool (MMAT) (version 2018), developed by Pierre Pluye et al. at McGill University, Montreal, Canada, which is suitable for assessing studies with diverse designs. This approach ensured a consistent and appropriate evaluation across all study types. For Randomized Controlled Trials (RCTs), the following aspects were assessed: proper randomization, baseline comparability, completeness of outcome data, blinding of outcome assessment, and adherence to the intervention. For non-RCTs, the following aspects were assessed: representativeness of participants, appropriate measurement of outcomes and exposure, completeness of outcome data, control of confounders, and whether the intervention was delivered as intended. Studies were classified as low risk of bias when most quality criteria were met and no major methodological concerns were identified. Studies were considered moderate risk of bias when some criteria were not fully satisfied but the study did not have significant limitations that could compromise the validity of the findings.

The detailed results of the quality assessment for each study are presented in Table 2 and Table 3.

### 2.4. Protocol and Registration

The protocol for this systematic review was developed a priori and preregistered on the Open Science Framework (OSF) on March 2026. The registration is currently under embargo. Upon publication of the manuscript, the registration will be made publicly accessible.

## 3. Results

### 3.1. Characteristics of the Included Studies

Table 4 summarizes the characteristics of the studies included in this review. The study sample in each of the articles varied from 10 to 342 women. A total of 2673 women were enlisted in the included studies. This systematic review included five quasi-experimental studies [26,27,29,30,31], 9 RCTs [17,18,19,20,21,22,23,24,25], two intervention studies [33,34], four experimental studies [28,35,36,37], one retrospective study [38] and one cross-sectional study [32].

### 3.2. Topical Natural, Herbal, and Biologically Based Treatments for the Prevention and Management of Nipple Trauma

Nipple trauma during early lactation is a frequent cause of pain, discomfort, and early discontinuation of breastfeeding. In this context, contemporary research has examined a range of topical pharmacological, herbal, and biological interventions aimed at both the prevention and treatment of nipple trauma. These include natural oils, expressed breast milk, lanolin, beeswax-based products, combination formulations, and nutritional approaches.

Evidence from primary studies indicates that several interventions can effectively reduce nipple pain and improve tissue healing. For example, the prenatal application of olive oil was associated with a significantly lower incidence of nipple trauma and pain compared to standard care (*p* ≤ 0.001) [26]. In the quasi-experimental study by Abdullah, Eshra, and Ashour (2022) [27], a significant reduction in nipple pain intensity was observed in both the group that applied expressed breast milk and the group that used peppermint water extract, compared to the control group, with the difference becoming particularly pronounced by day 14 (*p* ≤ 0.001). Comparable benefits were also reported in preventing nipple irritation in early postpartum women [31]. Similarly, randomized evidence suggests that topical olive oil may be more effective than expressed breast milk in reducing both pain and trauma severity during the early postpartum period [17]. Additional findings support the beneficial effects of coconut oil and olive oil in improving nipple condition compared to breast milk alone [29].

Lanolin is among the studied topical treatments. It has been shown to significantly reduce nipple pain and trauma over time [25]. However, comparative studies indicate that its effectiveness may be similar to that of expressed breast milk with no statistically significant differences between the two interventions [33]. Moreover, when combined with breastfeeding education, lanolin did not demonstrate additional preventive benefit compared to education alone [21].

Beeswax-based interventions and combination formulations have shown promising results. A randomized controlled trial demonstrated that a cream containing lanolin, beeswax, and olive oil significantly reduced pain and nipple injury compared to placebo with no reported adverse effects [20]. Likewise, the use of beeswax dressings was associated with lower rates of nipple pain and fissures compared to breast milk and dry care [24]. Additionally, beeswax breast pads combined with breastfeeding education were linked to improved breastfeeding continuation rates up to six months postpartum [22].

Nutritional interventions have also been explored. Daily consumption of carrot juice was associated with significant reductions in nipple fissures and duct blockage, which was possibly due to its antioxidant and anti-inflammatory properties [30].

Overall, topical interventions may help reduce pain and improve nipple condition, but none shows consistent superiority, and their effectiveness depends on proper breastfeeding technique.

### 3.3. Educational and Technical Breastfeeding Interventions as Strategies for Preventing Nipple Trauma

Educational and technical breastfeeding interventions play a central role in preventing nipple trauma, as they directly influence breastfeeding technique, maternal knowledge, and the level of professional and social support provided during the perinatal period. Evidence indicates that structured education—particularly when initiated antenatally and involving both the mother and her family—can significantly improve correct latch and reduce the incidence of nipple trauma in the early postpartum period [18].

Similarly, positioning-based approaches such as biological nurturing have been associated with a reduced risk of early breastfeeding-related breast problems, including nipple pain and fissures, although their effect on exclusive breastfeeding rates appears limited [19]. Interventions combining theoretical education with practical demonstration have consistently shown substantial improvements in breastfeeding technique; however, their direct impact on nipple trauma prevention is less consistent, suggesting that technique alone may not fully address the multifactorial etiology of nipple injury [36].

Educational programs have also been shown to significantly enhance maternal knowledge regarding breastfeeding practices and complication prevention, supporting behavior change and improved breastfeeding management [37]. Moreover, early postnatal targeted education has been linked to higher rates of exclusive breastfeeding and improved technical performance [34].

Overall, educational and technical interventions improve breastfeeding outcomes but are most effective when combined with broader clinical and psychosocial support.

### 3.4. Medical Devices and Interventional Approaches for the Management of Nipple Pain and Trauma

Medical device-based and interventional approaches represent alternative strategies for managing nipple pain and trauma, particularly in cases that do not respond to conservative or educational interventions. Overall, the evidence demonstrates heterogeneous effectiveness across different modalities.

The use of breast shells, a plastic devise with holes that fits over the nipple and allows ventilation, during the antenatal period did not significantly reduce the incidence of nipple pain or trauma compared to standard care, although it was associated with delayed onset of breast engorgement and high maternal satisfaction, suggesting a potential role in comfort rather than prevention [28].

Similarly, silicone nipple shields showed context-dependent effects. In mothers already using shields due to pain, milk transfer and percentage of available milk removed (PAMR) were not significantly affected. However, in dyads without prior need, shield introduction significantly reduced both milk volume and PAMR, indicating a decreased efficiency of milk removal. No additional pain reduction was observed with shield use [35].

In contrast, surgical nipple debridement for chronic, non-healing lesions demonstrated clear clinical benefits, including significantly higher rates of wound healing and pain relief compared to conservative management. However, recurrence rates remained similar between groups, suggesting that unresolved underlying causes may lead to relapse [38].

Regarding silver nipple cups, although a high proportion of mothers perceived them as effective, no significant differences were found in the incidence of nipple cracks or other lesions compared to non-users, indicating limited objective effectiveness despite high acceptability [32].

Photobiomodulation therapy (PBMT) did not demonstrate significant benefits in reducing nipple pain or improving quality of life compared to placebo; however, the extremely small sample size limits the reliability of these findings [23].

Interventional approaches may be effective in selected cases, whereas most device-based and technological interventions show limited or inconsistent benefits, supporting the need for individualized use and further high-quality research.

Overall, outcome measures varied considerably across studies. Pain was commonly assessed using visual analogue scales or numerical rating scales, while nipple trauma and healing were evaluated using a combination of clinical inspection tools and participant self-reports. In some studies, definitions of healing and severity of trauma were not standardized, further contributing to variability.

## 4. Discussion

This systematic review highlights that the prevention and management of nipple pain and trauma during breastfeeding is a clinically significant area, yet it is characterized by substantial heterogeneity in interventions, application protocols, and outcome measurement tools. The reviewed interventions can be functionally grouped into (i) herbal/biological and “traditional” topical applications, (ii) dermatological/pharmaceutical topical approaches, and (iii) medical device based and non-pharmacological interventional methods, including breastfeeding education, technique support, and professional guidance. In many studies, education on breastfeeding technique and professional support emerged as a critical factor.

Overall, while several interventions appear to provide relief or reduce the severity of nipple cracks, the evidence is mixed: some approaches show positive effects at specific time points or on selected outcomes, others do not outperform standard care or expressed breast milk, and in many cases, methodological limitations or lack of generalizability reduce the strength of conclusions.

Theoretically, the findings align with the contemporary view that nipple pain and trauma often result from repeated mechanical stress (traction, shear, friction) on the nipple–areola complex, causing microtrauma and inflammation, particularly when the “fit and hold” or lactation biomechanics are suboptimal. These factors are not consistently assessed or reported in the literature, which may contribute to variability in outcomes across studies evaluating interventions for nipple trauma. Additionally, overhydration and prolonged moisture (e.g., from pads, occlusive conditions, or inappropriate topical applications) may lead to moisture-associated skin damage (MASD), increasing epithelial vulnerability and delaying healing. This “mechanobiological” perspective helps explain why certain interventions fail when applied without addressing technique or when they exacerbate nipple moisture [5].

Regarding herbal/biological interventions, some studies evaluated olive oil as well as other oils or natural substances (e.g., coconut oil, beeswax, plant-based preparations) as preventive or therapeutic options. Overall, topical oily applications are often associated with improvement in subjective discomfort or reduction in nipple crack severity in some populations. However, protocols (frequency, duration, method) are inconsistent, and studies vary in baseline nipple condition, maternal education, and concurrent optimization of breastfeeding technique. Therefore, positive outcomes should be interpreted as “effectiveness within a specific care context” rather than as universally effective treatments. Evidence from a systematic review of specific plant-based interventions (e.g., Aloe vera) suggests that despite promising results, the evidence base is limited by small sample sizes, incomplete searches or selection, and absence of meta-analysis, warranting cautious clinical application [39]. Conversely, another systematic review suggests that the use of extra virgin olive oil may be effective in reducing nipple pain and trauma [16].

For dermatological/pharmaceutical interventions, lanolin was among the most studied options, sometimes as monotherapy, sometimes compared with breast milk or standard care, and occasionally combined with education or other supportive measures. A recent systematic review reported that the application of lanolin reduced significantly nipple pain and nipple trauma in lactating mothers [14]. However, findings suggest that lanolin may provide pain relief or improved healing in some trials, yet the overall international literature is inconsistent. Some data show no clear superiority over breast milk or no treatment, and there are theoretical and clinical concerns that occlusive or highly moisturizing applications may increase the risk of epithelial overhydration and MASD under certain conditions. Thus, lanolin should be used in a targeted manner within a framework that addresses mechanical causes and moisture management (e.g., frequent pad changes, avoiding prolonged occlusion) [5].

Εducational and technical interventions were found to improve breastfeeding outcomes, as also stated in the systematic review by Chagas et al. (2026) [16], but they were most effective when combined with broader clinical and psychosocial support. A key observation is that some interventions focus less on the “product” and more on lactation biomechanics and education/support. International evidence indicates that suboptimal latch and positioning are often primary causes of nipple pain/trauma; therefore, any topical treatment without correcting “fit and hold” may have limited or short-lived effectiveness. This insight helps unify the findings: where structured technique support was provided, outcomes were generally more favorable or stable, whereas without sufficient technique control, the effect of topical applications is harder to attribute causally [5].

The section on medical device and interventional approaches presents an even more complex picture. The prenatal use of protective breast shells did not clearly or significantly reduce pain or trauma frequency, although some evidence suggested potential benefits for comfort or congestion timing. Similarly, nipple shields showed variable effects on milk transfer: in dyads already using shields due to pain, no significant reduction in milk removal was observed, whereas in previously unshielded dyads, introduction was associated with reduced milk volume and PAMR, which is a clinically relevant finding affecting milk removal efficiency and potentially breastfeeding. Surgical debridement for chronic trauma demonstrated clear superiority in healing rates and pain relief in retrospective designs, but recurrence rates did not differ significantly, supporting the idea that while the intervention restores a healing environment, persistent mechanical or etiological factors may lead to recurrence. Silver cups were primarily evaluated observationally, showing high perceived effectiveness but no clear superiority regarding nipple cracks, suggesting possible placebo effects or selective use and highlighting the need for stronger randomized trials. Photobiomodulation therapy (PBMT) did not show clear analgesic superiority, but the very small sample size limits statistical power; thus, the lack of effect should be interpreted as “insufficient evidence” rather than definitive ineffectiveness.

Three factors explain why strong, hierarchical recommendations are difficult to derive. First, there is considerable clinical and methodological heterogeneity: different assessment time points (early postpartum days vs. weeks), varying definitions of trauma (mild sensitivity vs. cracks/bleeding), diverse pain scales, and often unstandardized “usual care.” Second, isolating the intervention effect from concurrent technique improvements or behavioral changes (e.g., feeding frequency, pump use, pad application, positional adjustments) is challenging. Third, “omitted variable bias” affects nipple pain literature: while correct latch is clinically primary, biomechanical parameters are often unmeasured or poorly described, structurally limiting inter-study comparisons [39].

Clinically, the application of findings should be algorithmic and etiology-based rather than “product-centered.” The first principle is the immediate assessment of breastfeeding (position, latch, milk transfer effectiveness, compression/ friction points, possible oversupply, pump use, correct flange size). Minimizing repeated mechanical microtrauma is the central lever for prevention and therapy. The second principle is careful moisture management: prolonged occlusion or moisturizing/hydrogel applications may worsen MASD in a vulnerable epithelium; individualized approaches, frequent pad changes, and avoidance of prolonged moisture are recommended. The third principle is that topical applications (e.g., olive oil, lanolin, breast milk) may serve as adjunctive options but cannot replace technique correction and the management of triggering factors [5].

A practical clinical framework can be proposed: (1) rapid assessment and correction of fit and hold, (2) differential diagnosis in persistent pain (e.g., vasospasm/Raynaud, contact dermatitis, pump trauma, bacterial infection/cellulitis if indicated), (3) mild–moderate damage: simple low-risk adjunctive care (breast milk or appropriate lubricant use with moisture precautions), (4) suspected pump-related exacerbation: check flange size/vacuum/duration, avoid friction, (5) chronic or persistent damage unresponsive to etiologic correction: referral to a specialized unit, where more invasive options (e.g., chronic necrotic/keratinized tissue debridement) may be considered with clear explanation that recurrence is possible if mechanical triggers persist [5].

Notably, only a limited number of included primary studies directly evaluated breastfeeding duration or continuation as a primary outcome. For example, Pastor-Pagés et al. (2025) [22] showed higher breastfeeding continuation at 3 and 6 months in mothers using beeswax pads combined with education, while Souza et al. (2020) [34] found educational intervention on correct technique significantly increased exclusive breastfeeding in the first month postpartum. In contrast, biological nurturing approaches [19] did not significantly alter exclusive breastfeeding rates in follow-up months despite reduced early breast problems. Most studies still focus on short-term clinical outcomes, such as nipple pain intensity, the presence of cracks or trauma, healing speed, and maternal comfort, leaving the long-term impact on breastfeeding continuation only partially and indirectly documented.

This highlights a crucial research gap: breastfeeding duration is a key maternal and neonatal health indicator and central goal of lactation support interventions. Future studies should include long-term follow-up and clearly defined outcomes related to exclusive and total breastfeeding to clarify the true clinical significance of interventions targeting nipple trauma prevention and management.

Also, as far as it concerns outcome measurements of the included studies, they varied considerably across studies. Pain was commonly assessed using visual analogue scales or numerical rating scales, while nipple trauma and healing were evaluated using a combination of clinical inspection tools and participant self-reports. In some studies, definitions of healing and severity of trauma were not standardized, further contributing to variability.

The review also identifies research gaps for future study. First, well-designed RCTs are needed to compare topical interventions under strict breastfeeding technique control (e.g., standardized lactation support as common background) to isolate the product’s effect. Second, outcome standardization is needed: common pain tools (e.g., VAS at set time points), uniform nipple trauma definitions/grading, and the inclusion of functional outcomes such as exclusive breastfeeding, duration, and supplementation needs. Third, studies should explicitly examine moisture management (MASD), e.g., “dry wound strategy” versus hydrogel/occlusive pads with parallel measurement of skin markers and microbiome. Fourth, medical device interventions (silver cups, breast shells, PBMT) require randomized trials with sufficient power, as current evidence is often observational or small-scale. Fifth, for chronic lesions, “step-up” protocols (technique + moisture management first, then targeted topical therapies, and finally interventional options) should be evaluated for healing and recurrence prevention [5,15].

In summary, based on recent evidence included in this review (2020–2026), no single intervention appears to be consistently superior for the management of nipple pain and trauma. Effectiveness depends on addressing the primary mechanical cause, protecting skin integrity (particularly against overhydration/MASD), and providing timely, individualized support. Topical products and devices can play a role but function best as adjuncts within a framework of correct technique and proper nipple care. Clinically, an etiology-based, stepwise algorithm is more beneficial than reliance on a single local therapy.

### Limitations of the Review

This review has several limitations. The literature search was restricted to the last 5.5 years (2020–2026), which may have excluded earlier studies and limited the overall comprehensiveness of the evidence. This decision was made to reflect current clinical practice; however, it may contribute to the variability of findings.

Suboptimal latch or ineffective sucking mechanics may increase mechanical stress on the nipple, leading to tissue damage and impaired healing. These factors are not consistently assessed or reported in the literature, which may contribute to variability in outcomes across studies evaluating interventions for nipple trauma.

## 5. Conclusions

Nipple pain and trauma during lactation are multifactorial phenomena with significant impact on breastfeeding continuation and quality. The synthesis of available evidence within the included studies indicates that no single therapeutic approach demonstrates consistent superiority. Instead, a range of interventions of varying types and effectiveness exists, with clinical value largely depending on the etiology, severity, chronicity of the injury, and the level of support provided to the breastfeeding mother.

A key contribution of this review is the development of an etiology-based, stepwise clinical framework that integrates early correction of breastfeeding technique, maintenance of nipple skin integrity, and ongoing professional and psychosocial support with targeted use of additional interventions in persistent cases. This framework emphasizes identifying underlying causes first, which is followed by targeted and individualized management rather than routine reliance on isolated treatments.

Despite substantial research activity in this field, the available evidence is characterized by methodological heterogeneity, small sample sizes, and a limited comparability of outcomes, which restricts the formulation of strong clinical guidelines. Future well-designed randomized controlled trials with standardized intervention protocols and consistent evaluation tools are needed to identify the most effective prevention and treatment strategies.

## Figures and Tables

**Table 1:** PICO framework.

PICO Element
Population (P): Breastfeeding women, regardless of age, mode of delivery, or care setting (hospital or community), with the presence or risk of nipple trauma.
Intervention (I): Interventions aimed at the prevention and/or management of nipple trauma during breastfeeding.
Comparison (C): Usual care, no intervention, placebo, or alternative interventions.
Outcomes (O): Outcomes related to pain intensity, improvement or healing of nipple trauma, and/or breastfeeding continuation or duration.

**Table 2:** Summary of the quality appraisal of randomized controlled trials using the MMAT (Mixed Methods Appraisal Tool).

Quantitative Randomized Controlled Trials	Methodological Quality Criteria					
	Is randomization appropriately performed?	Are the groups comparable at baseline?	Are there complete outcome data?	Are outcome assessors blinded to the intervention provided?	Did the participants adhere to the assigned intervention?	Risk of Bias
Abdelmawgoud et al. [17]	Ν	Y	Y	N	Y	Moderate
Gao et al. [18]	Y	Y	Y	CT	Y	Low
Milinco et al. [19]	Y	Y	Y	CT	Y	Low
Modarresy et al. [20]	Y	Y	Y	Y	Y	Low
Oliveira et al. [21]	Y	Y	Y	N	Y	Low
Pagés et al. [22]	Y	Y	Y	N	Y	Low
Ralph et al. [23]	Y	Y	Y	Y	Y	Low
Serhatlioglu et.al [24]	Y	Y	Y	N	Y	Low
Shahnazi et al [25]	Y	Y	Y	Y	Y	Low

Y: Yes (if it met quality criterion), N: No (if it did not meet quality criterion), CT: cannot tell (if did not mention relevant information).

**Table 3:** Summary of the quality appraisal of non-randomized studies using the MMAT (Mixed Methods Appraisal Tool).

Quantitative Non-Randomized Studies	Methodological Quality Criteria					
	Are the participants representative of the target population?	Are measurements appropriate regarding both the outcome and intervention (or exposure)?	Are there complete outcome data?	Are the confounders accounted for in the design and analysis?	During the study period, is the intervention administered (or exposure occurred) as intended	Risk of Bias
Abd-Elfattah et al. [26]	Ν	Y	Y	N	Y	Moderate
Abdullah, Eshra and Ashour [27]	N	Y	Y	CT	CT	Moderate
Cecilio et al. [28]	Y	Y	Y	CT	Y	Low
Hables and Mahrous [29]	Y	Y	Y	CT	Y	Low
Mayasari, Amalia and Jayanti [30]	Y	Y	CT	CT	Y	Moderate
Rashid and Mir [31]	Y	Y	Y	N	Y	Low
Karabavir et al. [32]	Y	Y	CT	N	Y	Moderate
Mustafa, Hamedo and Mustafa [33]	Y	Y	Y	CT	Y	Low
Souza et al. [34]	Y	Y	Y	CT	Y	Low
Coentro et al [35]	Y	Y	Y	CT	Y	Low
Morais et al. [36]	Y	Y	Y	CT	Y	Low
Siregar [37]	Y	Y	Y	N	Y	Low
Gao et al. [38]	N	Y	Y	CT	Y	Moderate

Y: Yes (if it met quality criterion), N: No (if it did not meet quality criterion), CT: cannot tell (if did not mention relevant information).

**Table 4:** Summary of included studies.

	Title	First Author/Year/Country	Study Design /Sample Size/Time Point	Results Reducing Pain/ Promoting Healing	Improving Breastfeeding Experience/Breastfeeding Continuation or Duration
1	Effectiveness of Giving Carrot Juice (*Daucus carota* L.) in Overcoming Nipple Crack and Clogged Milk Duct in Breastfeeding Mother	Mayasari/2025/Indonesia [30]	Quasi-experimental study/15 breastfeeding women/3rd, 5th, 7th day postpartum	- Daily consumption of 250 mL of carrot juice for 7 days significantly reduced the severity of nipple fissures.	- It also significantly decreased the incidence of blocked milk ducts in breastfeeding mothers
2	Application of Olive Oil on Nipple in Late Pregnancy Period to Prevent Nipple Trauma during Lactation	Abd-Elwahab Abd-Elfattah/2022/Egypt [26]	Quasi-experimental study/120 primiparous women/7th day postpartum	Prenatal application of olive oil significantly reduced nipple trauma and pain one week postpartum compared to usual care.	- There was a dose-dependent relationship between the frequency and duration of use and improvements in outcomes.
3	A randomized controlled study on the effects of extra virgin olive oil compared to breast milk on painful and damaged nipples during lactation	Abdelmawgoud 2020/Egypt [17]	RCT/120 breastfeeding women/3rd, 7th, 14th day postpartum	- The topical application of extra virgin olive oil significantly reduced nipple pain and trauma. - It also accelerated healing compared with breast milk.	
4	Effect of olive oil, coconut oil, breast milk on nipple soreness among lactating mothers	Hables/2021/Egypt [29]	Quasi-experimental study/135 breastfeeding women/1st, 7th, 14th day postpartum	- All three interventions reduced nipple injury. - Olive oil was the most effective. - Coconut oil followed in effectiveness.	
5	Effect of Expressed Milk, Peppermint Water Versus Routine Care on Cracked Nipple among Lactating Women	Abdullah/2022/Egypt [27]	Quasi-experimental study/90 breastfeeding women/3rd, 7th, 14th day postpartum	- The topical application of expressed breast milk and an aqueous extract of mint significantly reduced nipple pain, sensitivity, and trauma compared with standard care. - Expressed breast milk demonstrated the greatest effectiveness and was associated with faster healing outcomes.	
6	Benefits of breast milk for prevention of sore nipple & association with their demographic variables: A quasi-experimental study	Rashid/2021/India [31]	Quasi-experimental study/70 breastfeeding women/3rd, 5th, 15th day postpartum	- Topical application of breast milk significantly reduced nipple irritation in postpartum women. - The effect was independent of most demographic characteristics.	
7	Effect of Lanolin Versus Breast Milk on Traumatic Nipples for Lactating Mothers	Mustafa/2021/Egypt [33]	Intervention study/100 breastfeeding women/7th, 14th day postpartum.	- Both lanolin and breast milk significantly reduced nipple pain and trauma There was no statistically significant difference in effectiveness between the two interventions.	
8	Efficacy of a Topical Lanolin–Beeswax–Olive Oil Blend (RepoGen Cream) in Preventing Nipple Fissures in Breastfeeding Women: A Randomized Controlled Trial	Modarresy/2025/Iran [20]	RCT/133 mother–infant dyads/3rd, 7th day postpartum	- Topical application of RepoGen significantly reduced nipple pain, trauma, redness, and bleeding. - No adverse effects were observed.	
9	Lanolin and prenatal health education for prevention of nipple pain and trauma: Randomized clinical trial	Oliveira/2021/Brazil [21]	RCT/66 pregnant women/prenatally and 8th day postpartum	- The use of anhydrous lanolin combined with prenatal education did not significantly reduce nipple pain or injury compared to education alone.	
10	The Effect of Lanolin Cream on Treatment of Traumatic Nipples in Breastfeeding Mothers: A Randomized Control Trial	Shahnazi/2025/Iran [25]	RCT/80 breastfeeding women/day 14 postpartum/3rd, 7th, 14th day postpartum	- Topical application of lanolin significantly reduced nipple pain and the degree of injury - It was more effective than placebo. - It is considered an effective and safe therapeutic intervention.	
11	Determination of the effectiveness of beeswax in preventing nipple pain and cracks in primiparous breastfeeding mothers: A randomized controlled trial	Serhatlioglu/2023/Turkey [24]	RCT/90 breastfeeding women/1st, 3rd, 5th, 7th, 10th day postpartum	- The use of a beeswax barrier significantly reduced nipple pain and fissures. - It was more effective than breast milk and standard care.	
12	Effects of prenatal professional breastfeeding education for the family	Gao/2022/China [18]	RCT/342 pregnant women/3rd day postpartum.	- Structured prenatal breastfeeding education with family involvement nearly halved the incidence of nipple injury.	- It significantly improved correct latch technique.
13	The influence of breastfeeding technique education on knowledge of lactation problem prevention	Siregar/2025/Indonesia [37]	Pre-experimental design/26 breastfeeding women/not reported		Breastfeeding technique education significantly increased postpartum mothers’ knowledge of preventing lactation problems (*p* < 0.05).
14	Effect of an educational intervention on the breastfeeding technique on the prevalence of exclusive breastfeeding	Souza/2020/Brazil [34]	Quasi-randomized intervention study/180 mother–infant dyads/2nd, 30th day postpartum		- Targeted education on proper breastfeeding technique significantly improved correct technique. - It also increased the rate of exclusive breastfeeding in the first month of life. - It reduced breastfeeding-related problems.
15	Effectiveness of biological nurturing on early breastfeeding problems: a randomized controlled trial	Milinco/2020/Italy [19]	RCT/208 breastfeeding women/at discharge, 7th day postpartum, 4th month	- The biological nurturing approach significantly reduced the risk of breast problems, particularly nipple fissures and nipple pain.	- It had no significant effect on exclusive breastfeeding rates up to 4 months.
16	Breastfeeding technique and the incidence of nipple traumas in puerperal women attended in a city hospital: intervention study	Morais/2020/Brazil [36]	Quasi-randomized intervention study/180 mother–infant dyads/first month postpartum	- The educational intervention did not statistically reduce the incidence of nipple injury	- It significantly improved correct breastfeeding technique.
17	Breast shells for pain and nipple injury prevention: A non-randomized clinical trial	Cecilio/2022/Brazil [28]	Experimental study/62 breastfeeding women/prenatally and 14th day postpartum	- The prenatal use of breast shells did not significantly reduce nipple pain or trauma.	- It was associated with a delay in breast engorgement. - It was also associated with high maternal satisfaction
18	Impact of Nipple Shield Use on Milk Transfer and Maternal Nipple Pain	Coentro/2021/Australia [35]	Experimental study/59 mother–infant dyads/up to 20 weeks postnatally	- In breastfeeding women with nipple pain who were already using nipple shields, their use did improve pain compared with breastfeeding without a shield.	In breastfeeding women with nipple pain who were already using nipple shields, their use did not reduce milk transfer or the percentage of available milk removed (PAMR) compared with breastfeeding without a shield.
19	The Effect of Silver Nipple Cups in Nipple Care: Insights from Breastfeeding Mothers	Karabayır/2025/Turkey [32]	Cross-sectional study/298 breastfeeding women/not reported	- Despite the high perceived effectiveness of silver cups among mothers (89.9%), no reduction in the incidence of nipple fissures was observed compared to non-use.	
20	Ecological Beeswax Breast Pads Promote Breastfeeding in First-Time Mothers from the Valencian Community (Spain): A Randomized Trial	Pastor-Pagés/2025/Spain [22]	RCT/122 breastfeeding women/7th day, 3rd, 6th month postpartum		- The use of beeswax dressings combined with breastfeeding education significantly increased exclusive breastfeeding rates at 3 and 6 months. - It also improved mothers’ perceived health status.
21	A retrospective analysis of debridement in the treatment of chronic injury of lactating nipples	Gao/2021/China [38]	Retrospective study/167 breastfeeding women/7th day postpartum	- In 167 cases of chronic nipple injury, surgical debridement for chronic trauma significantly increased complete healing (54.3% vs. 26.7%). - It also significantly improved complete pain relief (48.1% vs. 23.3%) within 1 week. - There was no difference in recurrence within 1 month.	
22	In postnatal women with nipple pain, does photobiomodulation therapy (PBMT) at 660 nm compared with sham PBMT reduce pain on breastfeeding? A case series during COVID-19	Ralph/2023/Australia [23]	RCT (ultimately presented as a case series due to a small sample size)/10 breastfeeding women/not reported	- The intervention with photobiomodulation therapy (PBMT) did not lead to a statistically significant reduction in nipple pain.	- It also did not improve quality of life compared to the control group.

## Data Availability

No new data were created or analyzed in this study. All data generated or analyzed during this study are included in this published article.

## Abbreviations

The following abbreviations are used in this manuscript:

TEWL	Transepidermal Water Loss
PICO framework	(Population–Intervention–Comparison–Outcomes)
RCT	Randomized Controlled Trial
PAMR	Percentage of Available Milk Removed
PBMT	Photobiomodulation Therapy
MASD	Moisture-Associated Skin Damage
